# Perception of Advertisements for Healthy Food on Social Media: Effect of Attitude on Consumers’ Response

**DOI:** 10.3390/ijerph17186463

**Published:** 2020-09-04

**Authors:** Pedro Cuesta-Valiño, Pablo Gutiérrez Rodríguez, Estela Núñez-Barriopedro

**Affiliations:** 1 Department of Economics and Business Management, University of Alcalá, 28802 Alcalá de Henares, Spain; pedro.cuesta@uah.es; 2 Department of Business Administration, University of León, 24071 León, Spain; pablo.gutierrez@unileon.es

**Keywords:** healthy food, social media advertising, attitude, intention, consumer response, utilitarian value, hedonic value, social networks

## Abstract

The growing concern for health is currently a global trend, so promoting healthy products is an opportunity that companies can exploit to differentiate their products in highly competitive markets. The purpose of this research is to examine the antecedents of social media advertising value and their consequences for attitudes to healthy food and intentions to consume it, in a representative sample of Spanish consumers. The theory of Ducoffe’s advertising value model was used as a conceptual framework for the antecedents of attitudes based on utilitarian and hedonic values. To achieve this objective, a descriptive cross-sectional study was carried out based on primary data from a survey of a representative sample of the Spanish population with 2023 valid questionnaires. The Partial Least Square (PLS) method was applied to test the hypothesized relationships and predictive variables. The result of this research allows us to determine which variables influence the consumer’s response, as measured by intention, motivated by the consumer’s attitude to the value of healthy food, as influenced by the advertising value on social networks. Furthermore, the findings show that, for advertising healthy food on social networks to be valuable, it must be credible and richly informative.

## 1. Introduction

Concern for the health of the population in general and of consumers in particular is a topic of great interest today [1]. Thus, the promotion of healthier foods is gaining visibility [2]. Labeling food as healthy therefore attracts potential consumers and is a way of creating loyalty among current consumers, which is why it has become a trend in the current packaged food market [3].

In the literature, a variety of terms are used to describe healthy food. In Japan, these are referred to as “foods for specific health uses”, so they can be considered to have a medical application. In China, the term “functional foods” refers to foods containing additives aimed at reducing body fat, aiding digestion and other body functions [1,4,5]. In Taiwan, “healthy food” refers to foods that contain a specific nutrient or produce certain health effects [2]. The latter term, namely “healthy food”, has been adopted for this research because it allows a wider range of foods to be analyzed and is generally accepted in the literature [2,6,7,8]. Using this term will therefore contribute to greater knowledge. There are some studies that try to present the consumer perception of healthy food. Croll et al. [9] found that healthy eating was perceived to involve moderation, balance and variety. In the same vein, another study found that it is considered healthy when consumers enjoy a balanced diet, eating at regular times and eating according to the food pyramid. Moreover, it was also established that eating food with preservatives or additives were considered unhealthy [10].

In recent decades, of the models used in the literature to analyze consumer behavior, it is the theory of planned behavior (TPB) model that has become most widely accepted [11,12]. On the one hand, Yazdanpanah, Forouzani and Hojjati [13] have expanded the TPB with several variables, such as moral values and self-identity, in order to examine the intention of Iranian students to buy organic food. On the other hand, Lorenz, Hartmann, and Simons [14] have used TPB to study consumer behavior regarding products with origin labels. In addition, Yadav and Pathak [15] have applied it to study the green purchasing behavior of consumers in a developing nation.

Wong, Hsu and Chen [16] have used extended TPB to investigate consumer attitudes and purchase intentions for suboptimal foods. In turn, Patch et al. [4] have used TPB to study the intentions of consumers of functional foods. Furthermore, O’Connor et al. [5] have investigated the willingness of potential consumers to try functional foods, by applying a version of TPB in which a measure of willingness to try has replaced the intention variable.

In terms of the theory of social adaptation, values are defined as a set of personal and social cognitions that allow individuals to adapt to an environment, guiding them in the way they act in a situation [17,18]. Previous research [19,20] affirms that values are subjective and build attitudes and behaviors and are formed through the social and psychological development of consumers.

An increasing number of academic research studies have involved using the value–attitude–behavior (VAB) model to analyze consumer behavior. Thus, Honkanen, Verplanken and Olsen [21] have considered environmental morality to be an antecedent to organic food purchasing behavior. In research by Kang, Jun and Arendt [8] the VAB model is applied to investigate the purchase intentions for a low-calorie diet. In addition, Jun and others [7] have used the model to analyze the effects of health value effects on healthy eating intentions. Additionally, Hsiao-Ping, Chun-Chieh, and Han-Shen [2] have studied the functional beverage consumption of young university students in Taiwan.

The novelty of this work is established on the basis of three aspects. Firstly, this work uses a well-known model on social media advertising value but on a novel topic, such as healthy food on social networks. Second, previous studies have made progress in describing partial models of consumer behavior regarding healthy food. However, one of the most original aspects of this work is its analysis of a complete model that examines a wide range of indicators that influence social media advertising value. Specifically, it is the first model to include eating value variables and intention. Finally, the inclusion of the effects of social media advertising value on consumer response, as measured by attitude and intention, as a moderating variable. Intention has an indirect partial mediating effect on consumer response. These new aspects contribute to the small development of knowledge about the operation of social media advertising value on the consumer response, not only through the attitude, but also through the intention in the field of the healthy food.

Another novelty of this study is the size and design of the sample. In many previous studies the sample size has been fewer than 500 people and designed for a young segment, namely students [22,23,24,25,26,27,28,29,30,31,32]. However, in the present work the sample is composed of a total of 2023 people and includes not only the young segment but also older segments, since social networks, although more extensively used by young people, are increasingly used by older people who are also concerned about healthy food. This research therefore contributes to knowledge about a wider spectrum of consumers.

The aim of this work is, therefore, to ascertain which variables influence the consumer’s response through intention motivated by his/her attitude to the value of healthy food, which in turn is motivated both by internal stimuli—the utilitarian value of eating and the hedonic value of eating, and by external stimuli through social media advertising value.

## 2. Methods

### 2.1. Research Framework

**Figure 1:**
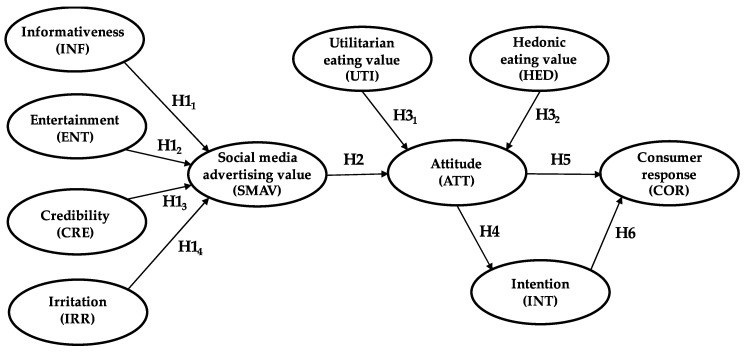
Model of Healthy Food Advertising on Social Media.

A new theoretical basis for understanding consumer responses is thus built, which is based on the value–attitude–behavior (VAB) model. Measurements of the Social Media Advertising Value (SMAV) variable are used to ascertain its relationship with attitudes and the latter’s relationships with consumer response and intention. Intention has an indirect partial mediating effect on consumer response. Finally, the values that determine attitude may be utilitarian or hedonic. This approach, with the hypotheses explained below, can be seen in the proposed model in Figure 1.

### 2.2. Research Hypotheses

The most commonly used theory to explain user perceptions and attitudes toward Internet advertising is the Ducoffe’s model [33]. Accordingly, Ducoffe [33] proposed informativeness, entertainment, credibility and irritation as antecedents of advertising value. The Social Media Advertising Value (SMAV) of healthy food refers to the individual subjective value of the potential consumer that, through online advertising, incorporates users’ interactions and any content they share. The advertising content may also include, with the consumer’s consent, various aspects of their person, such as photos, videos and names [28,31]. Some authors, when defining the SMAV, add the nuance of the relative subjective utility, to consumers, of the advertising [32,33,34,35,36,37]. Babin et al. [6] and Hamouda [38] add that the SMAV of healthy food affects consumer behavior through attitudes.

Previous studies, [22,26,28,29,30,39] have considered informativeness, entertainment, and credibility to be the antecedent variables of SMAV, and other authors [23,24,25,27,30,33,40,41,42] have also incorporated the irritation variable [26,33,41]. The present study considers all of these to be antecedent variables of the SMAV [38], in order to discover the extent to which each of these influences the SMAV of healthy foods. Informativeness in advertising is considered to be the ability to describe the characteristics and benefits of alternative products in a way that attempts to match consumer needs and desires by making the market more efficient [33,43]. Increasingly, consumers are looking for information on social networks supported by image or video messages [28]. Additionally, information from social media advertising may become viral [27], as consumers learn from the experience of other consumers by sharing information among their contacts. In this regard, Logan et al. [26] suggest that consumers in the social media environment are more receptive to reading advertising information. Informativeness in advertising thus creates a rational link between the brand and the consumer’s response. Many authors [22,23,24,25,26,27,28,29,30] consider that digital advertising should be informative, as a causal variable to social media advertising value. We can therefore propose the following hypothesis.

**Hypothesis** **1** **(H1_1_).** *Informativeness has a positive influence on the SMAV of healthy food.*

Entertainment is considered to be the ability to make pleasant, create fun and pleasure in the consumers [44]. On the other hand, Cheng et al. [41] define entertainment as how interesting the social media advertising is to the consumer. Because of the interactive style of social networks, users expect entertainment from their advertising content [38]. Likewise, entertainment in advertising creates an emotional link between the brand and the consumer’s response [45], so the user naturally plays the role of first evaluator of the advertising content in the network. There is a positive relationship between entertainment and social media advertising, which makes it a relevant factor to consider when designing an effective advertising campaign [27,28,46]. The more entertaining social media advertising is, the more value it will therefore have for the user, and consequently, the more interactions there will be in the consumer’s network with that advertising content. These interactions will include clicking “like”, writing comments and sharing the ad with contacts in the social network. The following hypothesis can therefore be established:

**Hypothesis** **1** **(H1_2_).** Entertainment has a positive influence on the SMAV of healthy food.

Credibility refers to how truthful or believable the consumers perceive the product information included in advertising content to be [47]. These types of reactions to advertising occur at the level of mental perception, and lead to the formation of trust or distrust in the advertising message [48]. Likewise, Logan et al. [26] establish that credibility is the expression of consumer expectations in relation to the reality of the advertising message. It is important to explain that the concept of credibility of advertising differs from “trusting beliefs”, since the former focuses on the information contained in advertising and the latter on people and organizations [49]. Therefore, credibility is an important element in having a positive influence on the value of advertising and, indirectly, on attitude [50]. Additionally, MacKenzie and Lutz [47] emphasize the importance of creating credible advertising to improve the effectiveness of advertising in creating value. In addition, the credibility of an advertising message in social networks is evaluated by network members through their comments [51]. Specifically, in the case of the credibility of advertising on social networks, this concept focuses on product information advertised in social networks rather than on trust in the platforms, links, websites or people who are managing the execution of that advertising [49]. Thus, the following hypothesis is proposed:

**Hypothesis** **1** **(H1_3_).** Credibility has a positive influence on the SMAV of healthy food.

While informativeness, entertainment and credibility are positive factors that increase social media advertising value, the irritation variable is a negative factor since it decreases SMAV [26].

Irritation can be considered as the way in which a consumer feels displeased for personal or social reasons [27] or due to the repetition of the ad, which causes saturation. Cheng et al. [41] explain how consumers can easily be irritated by digital advertising. They are, therefore, less likely to be persuaded by advertisements they consider annoying, offensive, manipulative, disappointing or dishonest, which thus generate less perceived value [26].

Some researchers consider that one of the main reasons why consumers tend to criticize advertising is irritation, which consequently leads to a reduction in advertising effectiveness [33].

In the specific case of social networks, Taylor, Lewin and Strutton [52] have found evidence of the possible negative impact of this variable on the SMAV and on the attitude of the advertisement’s audience. We therefore suggest the following hypothesis:

**Hypothesis** **1** **(H1_4_).** Irritation has a negative influence on the SMAV of healthy food.

The SMAV of healthy food refers to the individual subjective value of the potential consumer through online advertising that, with the consumer’s consent, incorporates the user’s interactions and what they post, and also shares different aspects of his or her person as photos, videos and names within the advertising content [28,31]. The SMAV generates consumer expectations [33], so it influences attitudes.

Attitude, on the other hand, is a feeling, which is a person’s learned predisposition to display a specific behavior or have a positive or negative thought [53]. It is an essential factor in predicting intention in consumer behavior [54].

In the context of advertising, Lutz [55] argues that attitude is the tendency to respond to a specific advertising stimulus during a specific occasion on which the advertisement is displayed. Luna-Nevarez and Torres [56], meanwhile, define attitude to advertising on social media as a predisposition to respond favorably or unfavorably to advertising on social networks.

In fact, attitude is affected by the perceived value of an advertisement [33]. A negative attitude generates the thought that advertising is intrusive and irritating [57]. Conversely, the more favorable the perception of the VAS, the more favorable the attitude [26,58,59,60]. The following hypothesis is therefore proposed:

**Hypothesis** **2** **(H2).** The SMAV of healthy food has a positive influence on attitude.

Healthy food provides benefits that are hedonic or utilitarian in nature [1,61,62]. Attitude has therefore been measured in this study from this double perspective, in which the hedonic eating value and the utilitarian eating value are considered to be the cognitive and affective antecedents, respectively, of attitude to this type of product.

According to Rodgers and Thorson [34], consumers can search online for information in advertisements for healthy foods, to obtain information that will meet their utilitarian needs. Utilitarian eating value is functional, goal-oriented and related to self-control [35].

In a virtual environment, social media advertising for healthy food can satisfy the consumer’s hedonic need by offering entertaining content [57]. Hedonic eating value is based on providing experiential consumption, which causes enjoyment, pleasure, happiness and/or fun [63]. Hedonic eating value is also produced by sensory attributes, such as the good taste of healthy foods [35].

According to the contributions of Voss et al. [36], the utilitarian eating value is made up of the functions provided by healthy foods, such as their nutritional composition and other health-related aspects, whereas the hedonic eating value is created by the experience of the product.

Utilitarian and hedonic eating values precede attitude, and establish “the most abstract level of knowledge, not being specific to the healthy food itself but influencing the perception and evaluation of it” (Tudoran; Scholderer; Brunsø) [6]. The following hypotheses are thus put forward:

**Hypothesis** **3** **(H3_1_).** The utilitarian eating value of healthy food has a positive influence on attitude.

**Hypothesis** **3** **(H3_2_).** The hedonic eating value of healthy food has a positive influence on attitude.

According to Rogers [64], intention is the most important and immediate indicator of people’s behavior, since “the assumption is that people do what they intend to do and do not do what they do not intend” Sheeran, [65] (p. 1). The intention is expressed in specific actions carried out on the basis of people’s decisions. They are identifiable through the form of responses that consumers make, using expressions such as “I intend, I expect or I plan”. Intentions are indicators of behavior but are also mediators of attitudes in their relationship to the behavioral response [65]. Attitude has therefore been identified as a factor influencing the prediction of the adoption of consumer intentions [66].

A range of social science research supports the role of attitudes in predicting behavioral intentions [67,68,69]. Additionally, the link between attitudes and adoption intentions is well-established in food research [1,4,5,70]. Consequently, one can expect that customers are likely to have intentions to eat healthy food as part of healthy eating, as long as they have first developed favorable attitudes. For all these reasons, the following hypothesis is put forward:

**Hypothesis** **4** **(H4).** Attitude in healthy food has a positive influence on intention.

Likewise, Luna-Nevarez and Torres [56] define attitude as a predisposition to respond positively or negatively to the advertising of healthy foods on social networks. It has even been argued that attitude is a determining factor in changing consumer behavior [71].

Likewise, attitude is affected by the perceived value of Social Media Advertising [22,33]. Thus, a negative attitude provokes an unfavorable consumer behavior response [57]. Conversely, the more favorable the attitude, the more favorable the consumer response [26,31,58,60]. Specifically, as favorable consumer responses, Wang and Sun [58] mention clicking on social media advertising to read more information or buying the product advertised on the social network, as a result of the favorable attitude encouraged by healthy food advertising [72]. Consumer response is therefore the result of consumer attitude [58,72,73]. Thus, the following hypothesis can be formulated:

**Hypothesis** **5** **(H5).** Attitude in healthy food has a positive influence on consumer response.

Previous studies [60] have highlighted that “consumers’ attitudes toward advertising influence their responses toward advertising, especially their intentions” (Mitchell and Olson) [74]. In the area of food, attitude often shows a strong association with intention [4,5,70,75]. In these studies, it was found that attitude was the most important determinant of purchase intent for functional foods.

This is in line with the premise of “the stronger the intention to perform a behavior, the more likely it is to be actually performed” Fishbein; Ajzen [53]. Additionally, Sheeran [65] shows that intention contributes moderately to explaining variations in actual behavior in the case of the consumption of healthy foods. Intention is a fundamental variable in consumer behavior, since the greater the intention the greater the possibility of generating a favorable consumer response in their behavior [53,76,77].

Thus, intention predicts healthy eating behavior [78,79]. The following hypothesis is therefore proposed.

**Hypothesis** **6** **(H6).** *Intention regarding healthy food has a positive influence on consumer response.*

### 2.3. Survey Design

This research is based on a cross-sectional descriptive study using primary data from a questionnaire answered by a representative sample of the Spanish population aged 15 to 69 during the month of June 2020. The total number of valid questionnaires collected was 2023, implying a sampling error of +/−2.22% (with a 95.5% confidence interval and *p* = *q* = 0.5) (see Table 1).

The questionnaire covers the 10 constructs of the model proposed (informativeness, entertainment, credibility, irritation, social media advertising value, utilitarian healthy eating value, hedonic healthy eating value, attitude, intention and consumer response), each with its different items (see Table 2). It also includes questions on a series of general classification variables (gender, age, educational level, family income and number of family members) and others specific to the use of social networks (social networks used and number of hours per day of social network use).

The initial selection of the different items of the 10 constructs of the questionnaire (see Table 2) was based on an exhaustive review of the existing literature. Once the items had been selected, and before sending out the questionnaire, prior qualitative research was carried out through a focus group. This focus group was comprised of four university professors from different Spanish universities with expertise in communication and healthy food and four professionals who worked in different food companies. As a result of this qualitative research, the final questionnaire was obtained, consisting of a total of 43 items.

With this questionnaire of 43 items, a pre-test was carried out in the month of April, over 3 days and on a representative sample of the Spanish population, made up of 40 persons between 15 and 69 years of age. The aim of this pre-test was to determine whether the scales were well constructed and to ensure that the people to whom the survey was to be addressed understood each of the questions in the questionnaire perfectly. After this process, some typos were corrected, and all questions were validated. Once the questionnaire had been refined, the survey was launched online during the first two weeks of June 2020, using the C.A.W.I. (Computer Assisted Web Interviewing, Google, Inc., Mountain View, CA, USA) technique through a discretionary non-probabilistic sampling by quotas, with the aim of achieving a distribution of sexes, ages and education levels that is as similar as possible to that of the Spanish population. The questionnaire had 43 items and in most of them a five-point Likert response format was used, which respondents rated from 1 (“completely disagree”) to 5 (“completely agree”) with a simple table format. For this reason, respondents could answer the questionnaire in an average time of 5–6 min via smartphones, tablets or computers. The questionnaire was distributed through the main social networks, with different reminders to motivate potential respondents to respond. The result was that, within two weeks, a representative sample of the Spanish population between 15 and 69 years old had been surveyed, with a total of 2023 valid questionnaires.

### 2.4. Sample Size and Composition

The total sample size was 2023 individuals who perfectly represent the Spanish population. The composition of the sample was 47% male and 53% female. By age group, 20% were 15–19 years old, 38% were 20–39 years old, 24% were 40–54 years old and 17% were 55–69 years old. By educational level, 15% had only basic schooling, while 36% had completed intermediate studies and 49% had attended tertiary education. By number of family unit members, only 4% consisted of only one person, 14% had two members, 23% had three, 44% had four and 15% had five or more members. Finally, analyzing the sample by monthly family income: 5% had an income below EUR 1000; for 30% their income was EUR 1000–1999; for 31% EUR 2000–2999; 18% had an income of EUR 3000–3999, 9% between EUR 4000–4999 and 7% over EUR 5000 (Table 3).

If we now look at the regular use of social networking, 98% of the sample said they regularly use WhatsApp (Brian Acton, Jan Koum, Mountain View, CA, USA), 71% YouTube (Chad Hurley, Steve Chen, Jawed Karim, San Bruno, CA, USA), 65% Instagram (Kevin Systrom and Mike Krieger, Menlo Park, CA, USA), 47% Facebook (Mark Zuckerberg, Eduardo Saverin, Andrew McCollum, Dustin Moskovitz, Chris Hughes, Menlo Park, CA, USA), 35% Twitter (Jack Dorsey, Noah Glass, Biz Stone, Evan Williams, San Francisco, CA, USA), and 14% used other social media platforms. By hours of daily social media usage, 15% of the sample said they spent less than 1 h per day on social media, 27% spent 1–2 h, 28% spent 2–3 h, 21% spent 3–5 h, and 10% spent 5 h or more on social media. Table 3 provides descriptive statistics for the sample.

### 2.5. Statistical Analysis

The theoretical framework was analyzed using SmartPLS version 3 (SmartPLs GMBH, Bönningstedt, Germany) [80]. Partial least squares (PLS), a structural equation modeling (SEM) tool, was used to perform the analyses. SEM enables researchers to simultaneously examine the structural component (path model) and measurement component (factor model) in one model, and PLS allows this tool to be used when researchers have to work with a non-normal distribution and avoid the transformations of variables that could produce problems in the interpretation of the model. This tool is more flexible than other options when applying measurement scales used in different surveys that are included in a new model, for example an extension of an existing structural theory. Furthermore, the goal is to predict key target constructs for obtaining more information about decisions on future variables. It is advisable to use PLS-SEM in this situation [81]. After analyzing the theoretical concepts, we chose to establish reflective constructs. The main reasons are that the effects when items are removed do not affect the content validity, while changes in the constructs affect the underlying measures and the items are correlated.

## 3. Results

### 3.1. Measurement Model: Reliability and Validity

In order to assess the measure’s reliability, we examined how each item relates to the latent constructs (see Table 4). These latent constructs were constructed with reflective measures. [82] Falk and Miller (1992) proposed, as a rule of thumb, retaining manifest variables with loadings that exceed 0.55—i.e., 30% of the variance of the manifest variable is related to the component. All of the loadings exceed 0.72 for these items and load more highly on their own construct than on others. These results provide strong support for the reliability of the formative measures. Internal consistency was assessed using three measures: Cronbach’s alpha, composite reliability (CR) and the average variance extracted (AVE). Nunnally and Bernstein [83] suggested a high internal consistency when Cronbach’s alpha is higher than 0.7. This value is also recommended for CR, and a value at least equal to 0.5 is recommended for AVE. As shown in Table 4, all the coefficients of each set of reflective measures in the study exceed 0.80 (less in the case of entertainment, but Cronbach’s alpha and CR exceed 0.7 and AVE exceed 0.5).

Discriminant validity was assessed comparing the square root of the AVE (i.e., the diagonal in Table 5) with the correlations among the constructs (i.e., the off-diagonal elements in Table 5); the diagonal elements should be greater than the off-diagonal elements [86]. The square root of the AVE for all of the formative constructs exceeds 0.7, and each is greater than the correlation between the constructs. These statistics suggest that each construct relates more strongly to its own measures than to the measures of other constructs.

### 3.2. Structural Model: Goodness of Fit Statistics

Absolute fit indices indicate how well a model fits the sample data [87]. A goodness of fit test conducted on the theoretical framework yielded the following results, which lie within the acceptable limits. Standardized Root Mean Square Residual (SRMR) is defined as the standardized difference between the observed and the predicted correlation. The SRMR has been used to assess model fit in PLS-SEM analyses [88,89]. A value less than 0.10 (or 0.08 in a more conservative version) is considered to indicate a good fit to data [90]. For this model, SRMR is 0.062, suggesting a well-fitting model. To account for possible method variance among the entire set of items, the model included an additional factor on which all items could load. The RMSEA and its 90% confidence interval for the model were 0.052 and (0.050, 0.053), respectively. This study used predictions, but for testing the model structure studies consistent PLS-SEM (PLSc) was used. These PLSc estimates of common factor models have been designed to mimic CB-SEM [91]. If we compare the results of PLS-SEM and PLSc, the differences between the two model coefficients are less than 0.1.

The results of the model also suggest that the dimensions explain a large amount of variance in SMAV, attitude, intention and consumer response, with R2 values of 0.61, 0.61, 0.12 and 0.35, respectively. The Stone–Geisser (Q2) results for SMAV, attitude, intention and consumer response are 0.49, 0.44, 0.100 and 0.26, respectively, where values larger than zero indicate the model’s predictive relevance for the dimensions

### 3.3. Results of SEM

**Figure 2:**
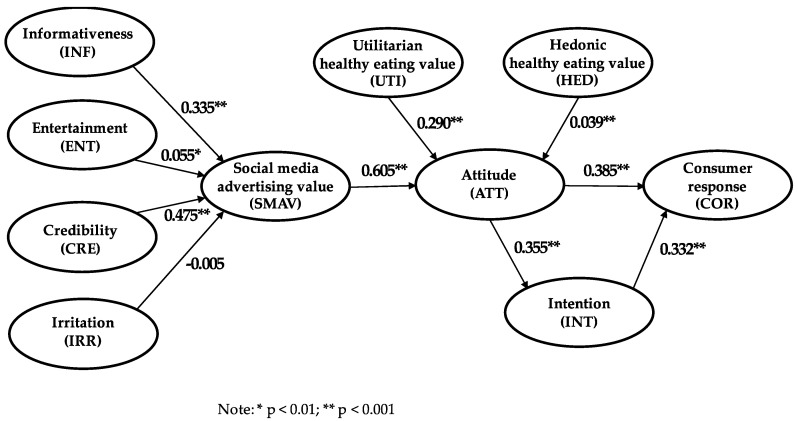
PLS-SEM results.

The conceptual model results (see Figure 2) show how each of the dimensions are related to the concept of SMAV. With a coefficient of 0.47, the results suggest that the credibility dimension of SMAV is the most important way to attempt to develop this variable. This is followed by the informativeness antecedent (0.34). The relationship between entertainment and SMAV indicates that entertainment has a very limited influence on SMAV (0.05). Finally, irritation does not have a significant influence on SMAV. We therefore do not reject H1_1_ or H1_3_ but do reject H1_2_ and H1_4_.

For the hypothesis attempting to discover the relationship between SMAV and attitude, it is very clear that the relationship is very strong, with a high coefficient (0.61). The other variables in the model that could influence attitudes are healthy eating values (utilitarian and hedonic). Both values have a positive and significant influence on attitude, but it is only the utilitarian healthy eating value that can have any impact on attitude (0.29) because the influence of hedonic healthy eating value is very low, as shown by its coefficient (0.04). Therefore, we fail to reject H2 and H3_1_ and reject H3_2_.

Finally, consumer response is subject to the moderating influence of two variables, attitude (0.39) and intention (0.33). However, intention has a partial mediating role between attitude and consumer response, and the influence of attitude on intention is relevant and significant (0.36). If this indirect effect is included, the total effect of attitude on consumer response is 0.50. We therefore do not reject H4, H5 or H6.

### 3.4. Prediction-Oriented Results

The PLS-SEM analyses provide information on the relative importance of constructs in explaining other constructs in the structural model (total effects) and also provide information on the scores for latent variables (and it is possible to use the mean). These results are particularly for prioritizing managerial actions. Table 6 provides the mean scores and total effects for latent variables.

## 4. Discussion

This research investigated whether the advertising value model can be applied to social media. The idea is to attempt to explain Spanish consumers’ responses to healthy food, incorporating measures of attitude formation and intention (see Table 7). This suggests that, although irritation is a factor in determining these consumers’ attitudes toward advertising value [30] and entertainment is usually a relevant antecedent of this dimension [26,28], the respondents assessed SMAV on the basis of informativeness and credibility. This suggests that, although social media advertising satisfies a consumer need for information exchange, this advertising is found to be more valuable when it is credible. The results from the sample indicate that credibility has the strongest effect on advertising value for social media ads. Informativeness had the second strongest effect on social media advertising value, with statistically significant effects. Furthermore, both antecedents have a moderate indirect effect on attitude toward social media advertising. Entertainment came in third in terms of the size of its effect on social media advertising value, but its relevance is very low. Lastly, irritation came out as the weakest predictor of SMAV in this study, failing to reach a significance level [26,30,41]. A possible explanation would be that the sample has clicked on social media advertising at some point, and for these, the path estimates for irritation came out as non-significant. In addition, we are focusing on healthy products, and we can say that, for this kind of product, the relationships between SMAV and its antecedents differ from the results found in the literature.

The path estimates for these variables (credibility and informativeness) are consistent with the results from the studies of social media advertising [22,26,30,33,38]. However, in the case of entertainment, the influence on SMAV is very weak. The most probable explanation is that this is due to the nature of the products selected for this study, which are healthy products. The consumers in question must decide to consume products that are important for their health and it is inevitable that the SMAV of healthy products is determined by aspects such as credibility and informativeness and not by entertainment or irritation. Thus, in the case of healthy food, the purchase is rational rather than emotional. As a result, the more information that is provided by the publicity for this kind of healthy product, and the more credible it is, the greater the effect is on the value of such advertisements for social network users [27]. This is especially true when the benefits of those foods are emphasized, and when they are compared with other foods.

Importantly, SMAV has a strong relationship with attitude to advertising. This finding suggests that this perceived value of advertising ultimately plays a greater role in the formulation of attitudes to advertising on social media. Within the study sample, perceived levels of informativeness and credibility—which are antecedents to SMAV—can predict, to some extent, the consumer’s attitude toward advertising.

Consumers were found to appreciate healthy eating values (both hedonic and utilitarian). Examining their simultaneous influence on attitude suggests that utilitarian healthy eating values apply a strong positive influence on a consumer’s attitude towards eating healthy food. The hedonic healthy eating value shows a very weak positive influence on attitude. This result coincides with the previous concept of healthy food, where consumers aim to find healthy and nutritional food as part of their decision-making process. It is also consistent with the results from other studies on hedonic and eating values [1].

Furthermore, there are two factors that affect the acceptance of advertising on social media, which could lead to more positive behavioral responses to advertising. Attitude and intention show important positive relationships to consumer response. Intention has a partial mediating role between attitude and consumer response, and this means that attitude has a strong total effect on consumer response. Several studies analyze a positive effect of attitude on consumer response, either in a direct relationship or through intention [38,60,85,92].

Accordingly, practitioners should note that effective advertising should vary the style and content of the message when they are advertising healthy food. Credibility, informativeness and entertainment value are key attributes used when assessing the advertising value of this food on social media. It is important to provide content that engages through credibility, rather than information. Not only does credibility have a greater influence on SMAV than informativeness, the credibility value score is higher than the score for informativeness and there is a greater choice of ways to develop this variable. Additionally, credibility is in the very nature of social media, because social media, and even social networks, are the most credible means of getting information to consumers. Campaigns to promote healthy food should therefore aim to show videos about the brand, or related images, over health-oriented services or news written by journalists and media professionals. However, even traditional banner advertising could attract this kind of consumer if we are focused on credibility and information.

If the final goal is to increase consumer response, attitude has been proven to be a good and significant option. SMAV has a strong relationship to attitude, and the attitude value score is not very high, but it is not the only way. The utilitarian value of healthy eating shows a significant influence on attitude to this type of food. This can assist advertisers in producing a good and credible informative message, because attitude and the utilitarian value of healthy eating are the most important antecedents of SMAV. Specifically, consumers are concerned about their weight and health issues. Perhaps this information is more relevant to attempting to create a suitable message, because this utilitarian eating value for healthy food scores very highly and it is going to be difficult to develop a mid-term strategy based on this eating value.

Finally, practitioners should understand the importance of intention on consumer response. However, its antecedent is attitude and, as with the last variable, its score value is very high. In any case, it is possible to find other sources for getting message content into social media advertising for healthy food, because consumers who intend, expect or plan to eat healthy food regularly are more likely to respond well to advertising for these products. Social media advertising should be used to achieve these specified goals.

Nevertheless, this study suffers from certain limitations. First, the sample was limited to Spanish consumers, and it is difficult to generalize the findings. Second, the survey was focused on different forms of advertising for different social media and the results could be improved by including a distinction between different forms of advertising on social media. Third, healthy food is the component being studied, and there is a broad diversity of healthy foods. Future studies could therefore explore the different forms of advertising and attempt to identify more clearly the consumers’ perception of what constitutes a healthy food. Additionally, a future study could attempt to obtain the results for healthy food by product categories. Integrating classification variables could be interesting, in order to enrich the proposed model and reveal the differences between groups. Alternatively, a study of mediator variables could also be a potential path for future research.

## 5. Conclusions

The current research contributes to the existing literature in that it provides empirical evidence of the capabilities of an extended model of the antecedents of SMAV, and the consequences for consumer response through attitude and intention. A conceptual model was proposed and empirically tested in the context of healthy food advertising on social networks.

The results of this research point out that credibility and information are key factors that add value to advertising in the context of healthy food and provide a great contribution to both literature and professionals in the sector. Firstly, credibility has a strong and positive influence on SMAV [26,47,49,51]. Secondly, informativeness also has a strong and positive influence on SMAV [22,23,24,25,26,27,28,29,30]. Likewise, both constructs have a moderate, indirect and positive influence on attitude toward social media advertising [50].

The fact that credibility and informativeness have a strong and positive effect on advertising value for social media ads, which implies greater certainty for the consumer of healthy food and therefore a reduction in the risk of misinformation on the Internet, especially on social networks, since it underlies the very interactivity of the receiver–producer–diffuser trinomial—prosume—with which online information is implemented, making it very difficult for many subjects lacking digital, informational and communicational skills to distinguish between information, rumors and disinformation [93], so this type of study makes it possible to contribute to media literacy, which is becoming increasingly important.

In relation to professionals in the advertising field, this contribution involves generating valuable advertising by designing advertisements that provide consumers with credible information, which generates more effective campaigns that positively influence consumer behavior.

In other types of consumer products, the entertainment construct has a positive influence on the generation of advertising value [27,28,38,46] and irritation has a negative influence [26,27,33,41]. In contrast, in the context of healthy food its influence is very low. Therefore, the model, in addition to contributing to the literature, provides an understanding of the extent to which constructs influence the SMAV, and implies a better allocation of resources in the design of advertising campaigns, since there is a great diversity of advertising styles that can be adopted to construct the message and design of an advertisement on the fourth screen.

On the other hand, this work contributes to media literacy with ethical and practical implications, since, as previous research points out [94,95], the current model of information production, characterized by a global prosumer society, with a multitude of connection platforms and with a greater need to be “connected”, is creating an oversaturated information society. Therefore, this paper recommends the use of informative and credible content in advertising to a greater extent than advertising content that appeals to entertainment or irritation in order to create value and positively encourage consumer attitude, purchase intention and response.

Utilitarian healthy eating values apply a strong positive influence on a consumer’s attitude to eating healthy food [1,61,62]. However, hedonic healthy eating value shows a positive influence on attitude [57] but it is very weak. Likewise, this is in line with previous studies [94] which indicate that subjects reject a large part of the information they receive due to their inability to process it, and the problem lies in the individual’s capacity to catalogue, filter and conserve useful information and discard that which has no value in terms of usefulness.

Attitude and intention show important positive relationships to consumer response, along the lines of previous work [38,60,85,92].

The findings allow for enhanced campaigns in constantly challenging environments. Thus, these organizations, through the use of accurate marketing and communication tools, will not only deliver information, but will work to reach their chosen target audience and will also achieve customer relationships, generate dialogue and offer healthy products that are better suited to their individual and ever-changing (more utilitarian than hedonistic) needs. Encouraging users to share advertising campaign content, decreasing marketing costs and promoting sales raises social media networks [96,97]. Therefore, the implications of this work on the response of social network users is essential to design effective advertising campaigns. Social network users have the possibility to make communities, share information, discuss and debate issues, and become active content creators [98].

As concerns other management implications, it can be stated that knowing the crucial elements, such as credibility, informativeness and usefulness, that generate value in the advertising of healthy products in social networks among a wide range of options of advertising content, serves to guide companies to improve their digital communication campaigns, to improve their audience targeting and reduce traditional marketing expenses and, as previous studies point [99], to improve their brand value. Likewise, these types of companies that are aware of the consumer’s needs, avoid the saturation of information by offering potential consumers more useful, credible and less irritating campaigns that improve their attitudes, intentions and consumer responses towards a healthy food consumption culture. Therefore, the model proposed in this work allows for improving digital marketing campaigns with more efficient campaigns which improve sustainability and business growth by generating value in advertising content, intentions and attitudes which improves the favorable responses of the consumer towards healthy food brands which can improve their brand equity.

So, the findings show that, in the case of healthy food, for advertising on social networks to be valuable, it must be credible and richly informative. These characteristics will result in a positive attitude to such an advertisement, which will in turn directly generate a favorable consumer response and an indirect effect, through intention, on consumer response. Attitude, which has a strongly associated total effect on consumer response, is related to utilitarian healthy eating value, but is not associated with hedonic healthy eating value. Attitude was also shown to exert an important influence on intention, and this therefore assists in achieving a strong total effect on consumer response.

## Figures and Tables

**Table 1:** Technical data sheet.

Universe	Males and Females Aged 15–69
Geographical scope	Spain
Field work	June 2020
Sample	2023 valid surveys
Sample error	+/−2.22 with a 95.5% confidence level and *p* = *q* = 0.5
Technique	C.A.W.I. (Computer Assisted Web Interviewing)

**Table 2:** Items by construct.

Construct	Number of Items
Informativeness	4
Entertainment	4
Credibility	4
Irritation	5
Social media advertising value	3
Utilitarian healthy eating value	3
Hedonic healthy eating value	3
Attitude	3
Intention	3
Consumer response	4

**Table 3:** Sample information.

**Gender**	**%**	**Total 2023**
Male	47.0	951
Female	53.0	1072
**Age**	**%**	**Total 2023**
15–19	19.8	401
20–39	38.4	776
40–54	24.4	494
55–69	17.4	352
**Level of studies**	**%**	**Total 2023**
Primary education	14.9	301
Secondary education	36.0	728
Higher education	49.1	994
**Number of family members**	**%**	**Total 2023**
1	4.3	87
2	14.1	284
3	22.9	463
4	43.8	885
5 or more	15.0	303
**Monthly family income (EUR)**	**%**	**Total 2023**
Less than 1000	5.2	105
1000–1999	29.9	604
2000–2999	30.8	623
3000–3999	18.2	368
4000–4999	8.7	176
5000 or more	7.3	147
**Most used social networks**	**%**	**Total 2023**
WhatsApp	97.7	1977
YouTube	71.4	1444
Instagram	65.1	1316
Facebook	47.0	951
Twitter	35.4	716
Others	14.3	284
**Use of social networks (h)**	**%**	**Total 2023**
Less than 1	14.8	299
1–2	27.1	548
2–3	27.7	560
3–5	20.6	416
More than 5	9.9	200

**Table 4:** Constructs, items, factor loading, reliability, and validity.

Factor Loadings		Sources of Adoption
**Informativeness (INF)** RVM: Cronbach’s alpha: 0.83, AVE: 0.66, Composite reliability: 0.89		
Advertising makes product information immediately accessible	0.79	Cheng et al. [41]
Advertising is a convenient source of product information	0.86	Dao et al. [28]
Advertising supplies relevant product information/brands	0.82	Ducoffe [33]
Ad informs me of the latest products and information available on the market	0.77	Logan et al. [26]
**Entertainment (ENT)** RVM: Cronbach’s alpha: 0.77, AVE: 0.59, Composite reliability: 0.85		
Advertising usually makes people laugh and has great amusement value	0.77	Cheng et al. [41]
I take pleasure in thinking about what I see, hear or read in advertisements	0.72	Dao et al. [28]
Advertising tells me what people who share my lifestyle will buy and use	0.83	Pollay and Mittal [43]
Advertising is more interesting than the content of another media	0.75	Logan et al. [26]
**Credibility (CRE)** RVM: Cronbach’s alpha: 0.87, AVE: 0.72, Composite reliability: 0.91		
Advertisements are credible	0.78	Dao et al. [28]
Advertisements are trustworthy	0.87	MacKenzie and Lutz [47]
Advertisements are believable	0.89	Murillo and Merino [30]
Advertising is convincing	0.86	MacKenzie and Lutz [47]
**Irritation (IRR)** RVM: Cronbach’s alpha: 0.89, AVE: 0.68, Composite reliability: 0.91		
Advertising is irritating	0.88	Cheng et al. [41] Ducoffe [33] Logan et al. [26]
Advertising is confusing	0.79	
Advertising is deceptive	0.84	
Advertising is annoying	0.88	
Advertising is too insistent	0.72	
**Social media advertising value (SMAV)** RVM: Cronbach’s alpha: 0.88, AVE: 0.81, Composite reliability: 0.93		
Advertisements are useful	0.90	Ducoffe [33]
Advertisements are valuable	0.91	Zen and Huang [84]
Advertisements are important (information)	0.89	Dao et al. [28]
**Attitude (ATT)** RVM: Cronbach’s alpha: 0.81, AVE: 0.72, Composite reliability: 0.89		
Advertising helps me to find products/services that match my personality and interests	0.89	Alwitt and Prabhaker [85]
Advertising helps me know which brands have the features I am looking for	0.86	Murillo and Merino [30]
Advertising is a good way to learn about what products/services are available	0.80	Hamouda [38]
**Intention (INT)** RVM: Cronbach’s alpha: 0.87, AVE: 0.80, Composite reliability: 0.92		
I intend to eat healthy foods regularly	0.87	
I expect to eat healthy foods regularly	0.93	Nystrand and Olsen [1]
I plan to eat healthy foods regularly	0.88	
**Consumer response (COR)** RVM: Cronbach’s alpha: 0.89, AVE: 0.75, Composite reliability: 0.92		
I will click advertisements shown in this social media	0.88	Zeng and Huang [84] Boateng and Okoe [60] Hamouda [38]
I will pay attention to advertisements shown on a social media	0.90	
I will search for related information about advertisements shown in this social media	0.85	
I will buy a product/service advertised on a social media	0.83	
**Utilitarian eating values (UTI)** RVM: Cronbach’s alpha: 0.88, AVE: 0.81, Composite reliability: 0.93		
It is important to me that the foods I eat Do not increase my weight	0.91	
It is important to me that the foods I eat Help me to avoid health issues	0.86	Nystrand and Olsen [1]
It is important to me that the foods I eat Help me to control my weight	0.93	
**Hedonic eating values (HED)** RVM: Cronbach’s alpha: 0.84, AVE: 0.76, Composite reliability: 0.90		
It is important to me that the foods I eat Are fun to eat	0.78	
It is important to me that the foods I eat Provide me good sensory feelings	0.91	Nystrand and Olsen [1]
It is important to me that the foods I eat Are enjoyable to eat	0.92	

Note: RVM = Reliability and Validity Measures.

**Table 5:** Correlations and Square Root of the AVE of the First-Order Latent Construct.

Fornell-Larker Criterion for Discriminant Validity	ATT	COR	CRE	ENT	HED	INF	INT	IRR	SMAV	UTI
Attitude (ATT)	0.85									
Consumer response (COR)	0.50	0.87								
Credibility (CRE)	0.66	0.44	0.85							
Entertainment (ENT)	0.53	0.43	0.58	0.77						
Hedonic eating values (HED)	0.46	0.35	0.37	0.32	0.87					
Informativeness (INF)	0.69	0.47	0.69	0.60	0.45	0.81				
Intention (INT)	0.36	0.47	0.26	0.23	0.51	0.36	0.89			
Irritation (IRR)	−0.09	−0.11	−0.22	0.03	0.00	−0.09	0.04	0.82		
Social media advertising value (SMAV)	0.72	0.46	0.74	0.53	0.38	0.69	0.29	−0.13	0.90	
Utilitarian eating values (UTI)	0.53	0.37	0.35	0.27	0.67	0.45	0.59	0.02	0.35	0.90

**Table 6:** Latent variables—mean scores and total effects.

Latent Variables		Total Effects			
	Mean	SMAV	Attitude	Intention	Consumer Response
Informativeness (INF)	3.40	0.335	0.202	0.072	0.102
Entertainment (ENT)	2.98	0.055	0.034	0.012	0.017
Credibility (CRE)	3.12	0.475	0.288	0.102	0.145
Irritation (IRR)	2.57	−0.005 *	−0.003 *	−0.001 *	−0.002 *
Social media advertising value (SMAV)	3.22	-	0.605	0.215	0.305
Utilitarian eating values (UTI)	3.93	-	0.290	0.103	0.146
Hedonic eating values (HED)	3.85	-	0.039	0.014	0.020
Attitude (ATT)	3.31	-	-	0.355	0.503
Intention (INT)	3.84	-	-	-	0.332
Consumer response (COR)	3.04	-	-	-	-

* Note *p* > 0.05.

**Table 7:** Summary of hypothesis verification.

Hypothesis	Content	Verification
H1_1_	Informativeness has a positive influence on SMAV in healthy food	Supported
H1_2_	Entertainment has a positive influence on SMAV in healthy food in healthy food	Rejected
H1_3_	Credibility has a positive influence on SMAV in healthy food in healthy food	Supported
H1_4_	Irritation has a negative influence on SMAV in healthy food in healthy food	Rejected
H2	SMAV in healthy food has a positive influence on attitude	Supported
H3_1_	Utilitarian eating value in healthy food has a positive influence on attitude	Supported
H3_2_	Hedonic eating value in healthy food has a positive influence on attitude	Rejected
H4	Attitude in healthy food has a positive influence on intention	Supported
H5	Attitude in healthy food has a positive influence on Consumer response	Supported
H6	Intention regarding healthy food has a positive influence on Consumer response	Supported
